# Data-driven assisted real-time optimal control strategy of submerged arc furnace via intelligent energy terminals considering large-scale renewable energy utilization

**DOI:** 10.1038/s41598-024-56193-0

**Published:** 2024-03-07

**Authors:** Bowen Zheng, Mingming Pan, Qixin Liu, Xu Xu, Chang Liu, Xuchen Wang, Wen Chu, Shiming Tian, Jindou Yuan, Yuting Xu, Zishang Xu, Yongjun Li

**Affiliations:** 1 China Electric Power Research Institute Co., Ltd, Beijing, 100192 China; 2 School of Advanced Technology, Xi’an Jiaotong-Liverpool University, Suzhou, 215000 China; 3 China International Engineering Consulting Corporation Co., Ltd, Beijing, 100192 China

**Keywords:** Renewable energy, Electrical and electronic engineering

## Abstract

This study presents a data-driven assisted real-time optimization model which is an innovative approach to address the challenges posed by integrating Submerged Arc Furnace (SAF) systems with renewable energy sources, specifically photovoltaic (PV) and wind power, with modern intelligent energy terminals. Specifically, the proposed method is divided into two stages. The first stage is related to data-driven prediction for addressing local time-varying renewable energy and electricity market prices with predicted information, and the second stage uses an optimization model for real-time SAF dispatch. Connections between intelligent energy terminals, demand-side devices, and load management systems are established to enhance local renewable resource utilization. Additionally, mathematical formulations of the operating resistance in SAF are explored, and deep neuron networks are employed and modified for dynamic uncertainty prediction. The proposed approach is validated through a case study involving an intelligent energy terminal with a 12.5 MVA SAF system and 12 MW capacity renewable generators in an electricity market with fluctuating prices. The findings of this research underscore the efficacy of the proposed optimization model in reducing operational costs and enhancing the utilization of localized renewable energy generation. By integrating four distinct dissatisfaction coefficients into the optimization framework, we demonstrate the model's adaptability and efficiency. The application of the optimization strategy delineated herein results in the SAF system's profitability oscillating between $111 and $416 across various time intervals, contingent upon the coefficient settings. Remarkably, an aggregate daily loss recovery amounting to $1,906.84 can be realized during the optimization period. Such outcomes not only signify considerable economic advantages but also contribute to grid stability and the diminution of renewable energy curtailment, thereby underscoring the dual benefits of economic efficiency and sustainability in energy management practices.

## Introduction

In light of the increasingly severe energy crisis and the growing issue of global environmental pollution, there is a projected increase in the adoption of alternative energy sources within the power system, particularly photovoltaic (PV) and wind power. The increased dependence on these energy sources gives rise to fluctuations and unpredictability, presenting notable obstacles to maintaining stable frequency regulation in the power grid. As the reliance on conventional thermal power units diminishes, relying exclusively on the regulatory capacities of these units becomes inadequate progressively in mitigating the inherent swings associated with renewable energies. Nevertheless, implementing demand-side response strategies, specifically targeting significant industrial loads, presents a promising way to address these issues. Substantial energy-intensive industrial loads, such as electrolytic aluminium furnaces, submerged arc furnaces, and polysilicon production furnaces, possess many advantageous characteristics. These systems have significant capabilities, prolonged heat storage periods, remarkable control capabilities, and substantial customizable possibilities. The ability to modify their active power within brief timeframes does not harm their production processes, rendering them highly promising adaptable assets within the power ecosystem.

However, existing submerged arc furnace (SAF) system planning studies have ignored that SAFs combined with dis- distributed energy generation farms can peak and dissipate excess power. For instance, Reference^1^ delves into optimizing raw materials with high Al_2_O_3_ content and enhancing processes in the SAF for increased aluminium and silicon extraction. In Reference^2^, a 3D transient mathematical model is presented to simulate multiphase flow and thermochemical reactions within an industrial furnace. Reference^3^ applies computational fluid dynamics (CFD) and a 3D model to assess the operations of a SAF in ferronickel production. Reference^4^ explores the influence of varying weight percentages of biomass pellets on biocoke’s properties and its potential use in blast and SAFs. In Reference^5^, a focus is placed on how different raw materials impact the silicon yield and energy consumption in SAFs. Reference^6^ offers a thermodynamic analysis of high-carbon ferromanganese alloy production in a SAF, with slag fluxing strategies and a simulation model. Reference^7^ describes the carbothermic process for producing specific iron-silicon-aluminum alloys in industrial settings. Most of studies focus on modeling and optimizing SAFs for various metallurgical applications, including silicon and other metal alloys. Several of these studies emphasize the significance of efficient smelting processes, the comprehensive comprehension of charge materials, and their influence on energy consumption. Additionally, they high-light the importance of advanced computational models, including 3D mathematical models and CFD models, in augmenting these processes. Acknowledging the multifaceted nature of contemporary SAFs, it is essential to recognize that they are not merely conventional smelting instruments but complex systems that can be customized to enhance their overall performance. By employing creative tactics and incorporating research insights from the initial design and planning phases, the collective capacity of these furnaces can be significantly enhanced.

There are also studies in the electrical field that focus on current, such as Reference^8^, which presents a comprehensive framework merging electrical dynamics with heat, material transition, and chemical reactions in furnace operations. Reference^9^ offers an in-depth exploration of a tri-phase SAF’s current pathways, highlighting elements like electrode configurations and primary arcs. Reference^10^ introduces a cutting-edge dual particle swarm optimization technique to bolster the efficiency and precision of model parameter determinations. Meanwhile, Reference^11^ delves into how electrode placements impact the SAF’s electrical nuances, utilizing a tri-dimensional design in ANSYS Maxwell that factors in alternating current via an eddy current mechanism.

Reference^12^ introduces an energy-sharing model based on the Nash bargaining game between multi-microgrids, while Reference^13^ proposes a multi-constraints single objective capacity optimization method for utility-scale photovoltaic-electrolysis-battery (WPEB) systems. In Reference^14^, a deep reinforcement learning method is applied to address dynamic and stochastic optimization problems in long-range planning, considering various uncertainties and constraints. Furthermore, Reference^15^ suggests a novel control technique for optimizing hybrid renewable energy sources (HRES) through the utilization of a high-gain DC-to-DC converter topology. The current literature on optimizing SAF systems needs to be revised, especially when integrated with PV and wind energy. One prevalent approach employs intelligent energy terminals to adjust power levels based on electricity spot market prices^16^. However, while this method offers insights, it needs to holistically address the intricacies of SAF systems. Specifically, there are challenges when considering (i) the incentive compensation from the ancillary service market, which can significantly alter the system’s economics, and (ii) the penalties associated with discrepancies between the real power of the SAF and the clearing power in the ancillary service market. To address these complexities, advanced optimization techniques are required. Therefore, the paper aims to establish connections between intelligent energy terminals, demand-side devices, and load management systems to improve the utilization of local renewable resources.

In Reference^17^, introduces a pioneering time sequence analysis (TSA) method. This method stands out for its ability to facilitate chronological analysis spanning from the initial year to the ultimate target year. Meanwhile, Reference^18^ addresses the formidable challenge of handling the uncertainty associated with wind power over an extensive temporal horizon. Employing a robust optimization (RO) approach grounded in the budget uncertainty set, this study prioritizes both robustness and economic considerations. However, despite the valuable contributions of References^17^^,^^18^ in the realms of time sequence analysis (TSA) and robust optimization (RO) for wind power uncertainty, there exist notable research gaps that our study aims to address.

Contrary to the predominant focus on modeling and optimizing submerged arc furnaces (SAFs) for metallurgical applications in the provided literature, there is a growing need to integrate SAF systems with renewable energy sources such as photovoltaic (PV) and wind energy. While references 13 and 14 discuss approaches involving intelligent energy terminals and time sequence analysis (TSA), respectively, the current literature lacks a comprehensive examination of the challenges associated with the integration of SAFs and renewable energy. The mentioned references touch on the economic considerations, electricity spot market prices, and even compensation from the ancillary service market. However, there is a distinct absence of detailed discussions on the potential hurdles arising from the interaction between SAFs and renewable energy, such as the fluctuating nature of wind power and its impact on system economics. Additionally, there is a need for more advanced optimization techniques that can address complexities like incentive compensation and penalties associated with discrepancies in power levels. In light of this, future research should aim to bridge the gap between intelligent energy terminals, demand-side devices, and load management systems, offering a more holistic perspective on the utilization of local renewable resources in conjunction with SAF systems.

In this regard, this paper proposes an innovative approach to address the challenges posed by integrating SAF systems with renewable energy sources, specifically PV and wind power, with modern intelligent energy terminals. Specifically, the proposed method is divided into two stages. The first stage is related to data-driven-based prediction for addressing local time-varying renewable energy and electricity market prices with predicted information, and the second stage is to use an optimization model for real-time SAF dispatch. The main contributions of this work can be summarized as follows:

1. Unlike existing literature on optimizing SAF systems, this paper focuses on the mathematical model of SAF to establish a comprehensive real-time control strategy for SAF with intelligent energy terminals. By employing this real-time control strategy, the SAF model can be more accurately controlled.
2. Considering the uncertainties arising from intermitted renewable generation and electricity prices, the prediction model is used and revised to address the possible issues. The optimization results for renewable dispatch and SAF control obtained from the proposed model are more reasonable with the high-accuracy predicted information.

The proposed method is divided into two stages. The first stage is related to data-driven-based prediction for addressing the local time-varying renewable energy and electricity market price with predicted information, and the second stage uses an optimization model for real-time SAF dispatch. Meanwhile, connections are established between intelligent energy terminals, demand-side devices, and load management systems to improve the utilization level of local renewable energy.

## Connection of intelligent energy terminals

In most cases, these intelligent energy terminals are installed on the side of the clients. They can establish communication linkages with the master station and various energy measuring and control terminals, and they are primarily utilized in areas with strong public wireless communication networks and less electromagnetic interference. These areas typically include customer substations and power distribution rooms. To establish a secure connection, these terminals establish a connection with the master station via a vertical security authentication platform. Furthermore, the connections are made with a multitude of devices through a combination of wired and wireless hybrid networking, including:

1. The utilization of intelligent equipment incorporating communication interfaces, such as central air conditioning chillers, distributed power inverters, and electric boiler control units. These interfaces facilitate the monitoring of equipment status in real time and allow for the delivery of control instructions to manage the power states of the equipment effectively.
2. The customer’s on-site measuring devices, including advanced electrical meters placed by power grid corporations or power demand response implementation agencies. The measurement and control modules are responsible for monitoring both electrical and non-electrical factors.
3. Load switches, also known as circuit breakers, are utilized in non-intelligent equipment to enable direct manipulation of the power states of said equipment.

**Figure 1:**
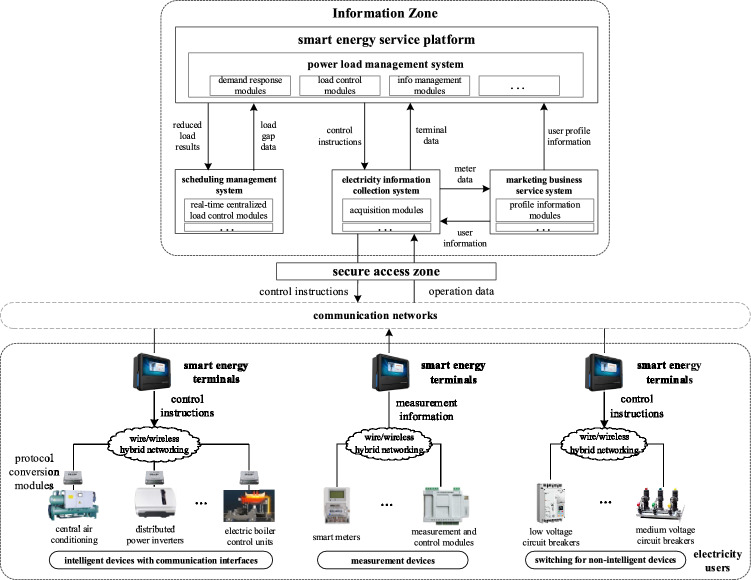
Connection between intelligent energy terminal and demand-side equipment and load management system.

Figure 1 depicts the interconnections of the intelligent energy terminals, the demand-side devices, as well the power loading administration system.

The intelligent energy terminals encompass a range of essential functionalities, including the acquisition of power data, the monitoring of power quality, and the evaluation of power usage. In addition, these systems offer additional functionalities such as resource management, flexible control, load monitoring, and the capacity to effectively coordinate and modify resource distribution.

In this study, we explore the critical roles that intelligent energy terminals play in the domain of electrical power systems. The aforementioned functions play a pivotal role in the attainment of effective energy management and the optimization of energy use. To provide a full overview of these functions, we have produced a table (see Table 1) that meticulously delineates essential areas such as resource management, flexible control, load monitoring, and changeable resource scheduling. The aforementioned features are achieved by implementing intelligent energy terminals, along with relevant technological solutions and strategic approaches. The next section provides a more comprehensive analysis of these functions, highlighting their inherent worth and pragmatic implementations within the realm of electrical power.

**Table 1:** Functional capabilities of intelligent energy terminals.

Function	Description
Resource management	Creation of a database for load resources, analysis of the characteristics of those resources, live monitoring, and identifying features
Flexible control	AI programs and control strategies can change the output at the equipment level, turning load control into a flexible interaction mode
Load monitoring	Tracking of energy use in real time, accurate control of electrical components, and help with load adjusting
Adjustable resource scheduling	Realization of adjustable resource access, load resource analysis, and scheduling for customer-side load control requirements

## Mathematical formulation

### Methodology description

**Figure 2:**
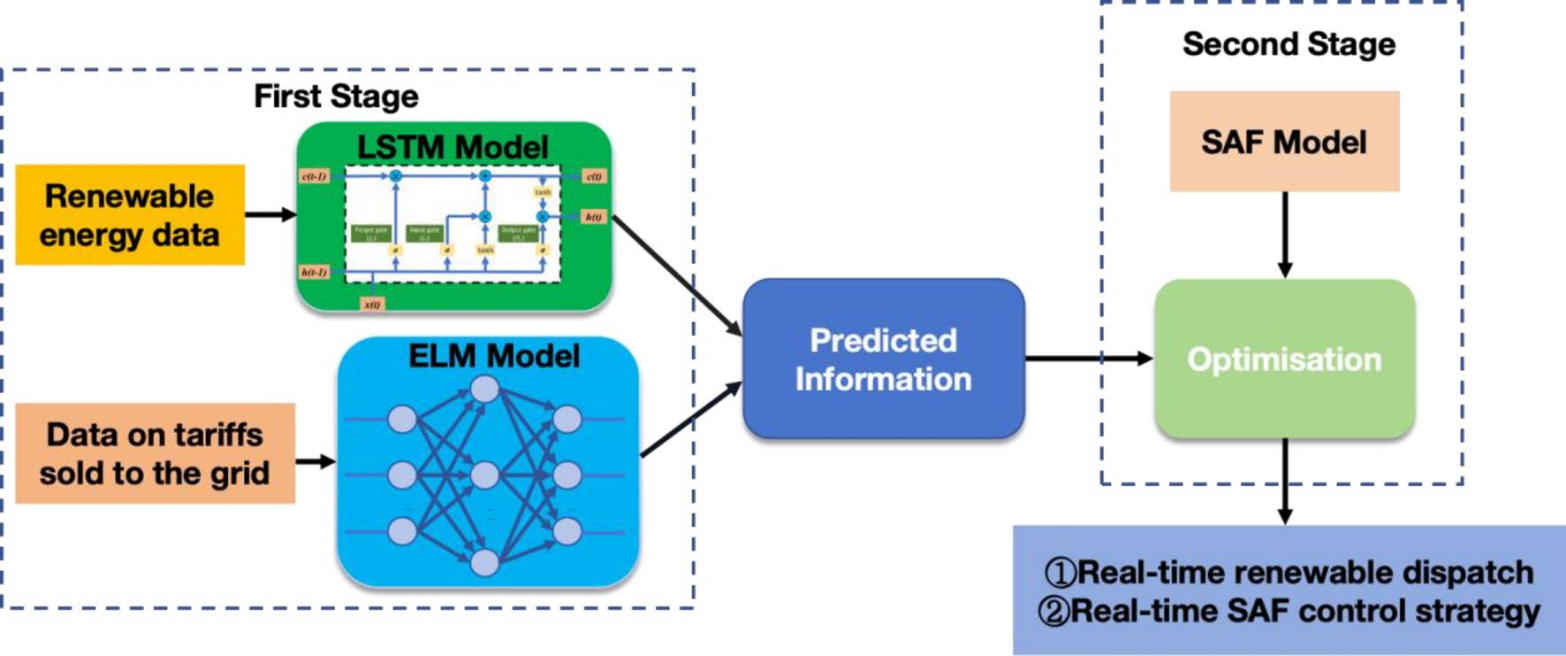
Two-stage data-driven assisted optimization model.

As shown in Fig. 2, this study employs a two-stage optimization model. In the first stage, sustainable energy data is processed through an LSTM encoder-decoder sequence-to-sequence model, successfully forecasting the power output of wind and solar energy. Concurrently, electricity price data for selling to the grid is processed using an ELM model, and the selling price is predicted using the ELM algorithm, thereby obtaining predictive information.

In the second stage, the SAF system is modeled and integrated with the intelligent energy control system proposed in this study. Within the SAF system, the working resistance is a critical parameter, and its mathematical model is incorporated into our study's model amidst a complex production process, encompassing factors such as resistance constants, current, and power. Following theoretical analysis and production evaluation, the mathematical relationship between the working resistance and the electrode insertion depth was established, leading to the derivation of the relationship between furnace power and electrode insertion depth. The optimization component of the second stage considers four main elements in the objective function: the profit margin between buying and selling electricity, dissatisfaction with the cost of electricity purchases, incentive compensation from the auxiliary service market, and SAF penalties due to differences in cleared power. Constraints include the relationship between surplus electricity and wind-solar output, surplus electricity and optimized electricity volume, the current operating power of the SAF and its total electricity sold, the relationship between SAF's baseline load and operating power, and the relationship between wind-solar power generation and electricity trading.

**Figure 3:**
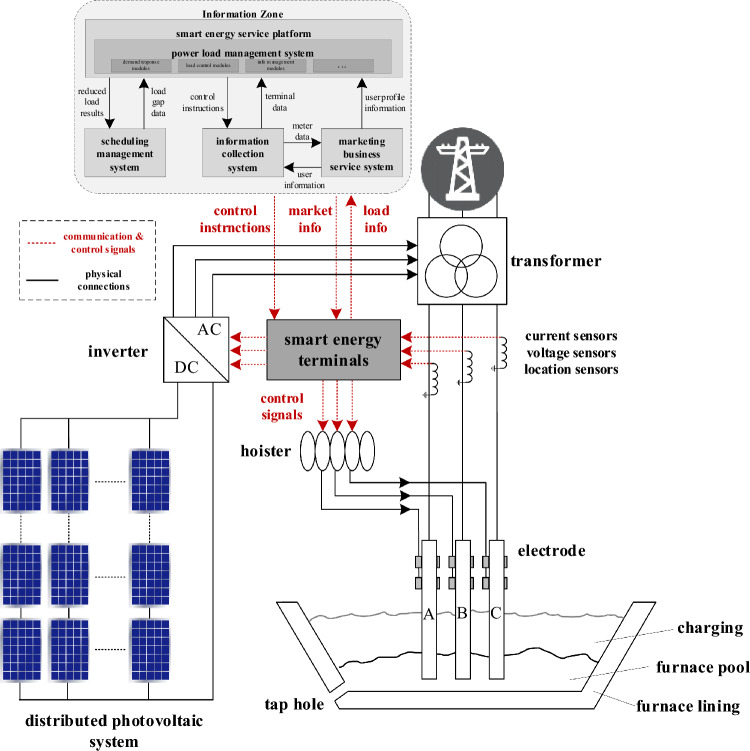
Intelligent energy terminal control system architecture for ferroalloy submerged arc furnace and distributed PV system.

Combining the optimizations of these two stages, we have obtained real-time sustainable energy dispatch results and SAF control strategies, providing reliable economic optimization for the overall sales direction of new energy and for SAF system strategies in response to market fluctuations (Fig. 3).

### Operating properties

In the ferroalloy submerged arc furnaces, operating resistance is one of the pivotal parameters. This is conventionally characterized as the resistance between the furnace's electrodes and its foundational bottom. The significance of this operating resistance cannot be understated, especially when considering the intricate production processes associated with these furnaces. Contemporary control strategies for ferroalloy submerged arc furnaces are predominantly anchored on the tenet that such resistance remains invariant. This is particularly evident when examining the resistance metrics associated with each phase electrode:

1 $$R={K}_{{\text{furnace}}}{P}_{{\text{furnace}}}^{-1/3}.$$

In the context where R represents the operating resistance of individual electrodes, ${{\text{K}}}_{{\text{furnace}}}$ signifies the product's resistance constant, and ${{\text{P}}}_{{\text{furance}}}$ denotes the cumulative effective power of the ferroalloy submerged arc furnaces. Given this,

2 $${P}_{{\text{furnace}}}=3{I}^{2}R.$$

Thus, we can get another expression equation for the total effective power of the SAF:

3 $${P}_{{\text{furnace}}}=2.28{I}^{1.5}{K}_{{\text{furnace}}}^{0.75}.$$

**Figure 4:**
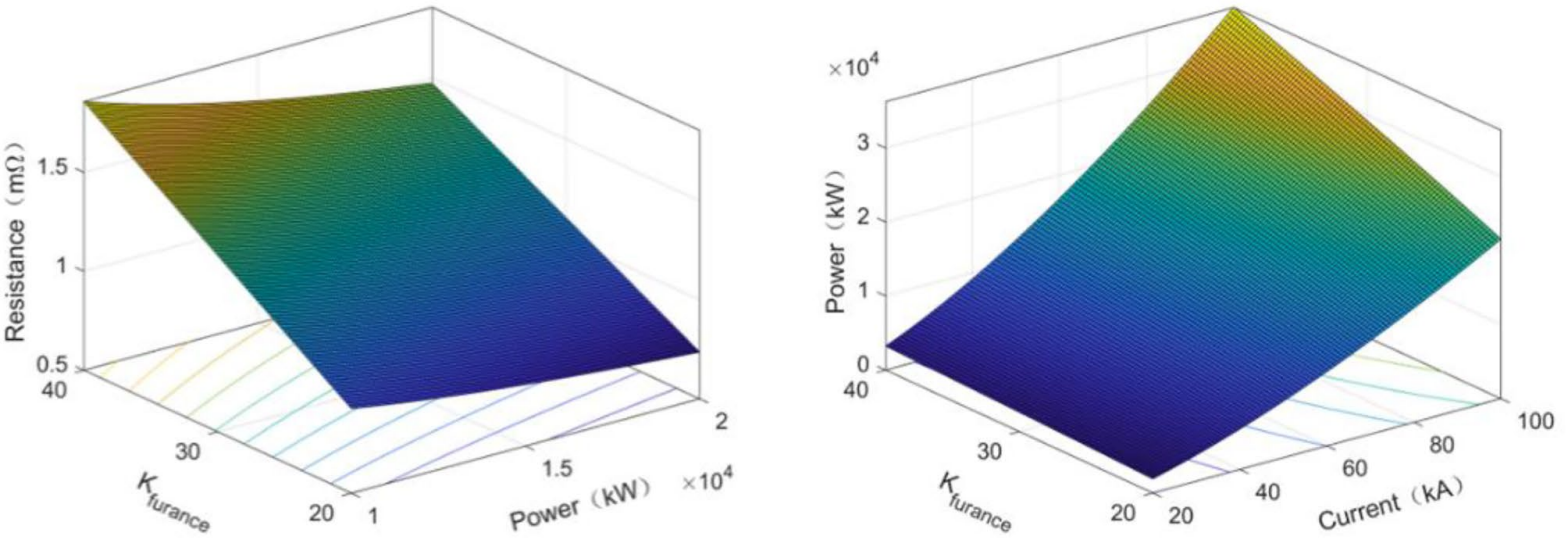
Ferroalloy submerged arc furnace operating resistance, resistance constant, current and power relations.

In the given context, I symbolizes the electrode current for each phase. The interrelationships between the operating resistance of the ferroalloy submerged arc furnace, its resistance constant, current, and power dynamics are illustrated in Fig. 4.

Both theoretical evaluations and practical production insights have confirmed that, given consistent geometric and electrical parameters, along with charging attributes of the ferroalloy submerged arc furnaces, an inverse relationship exists between the operating resistance of these furnaces and the depth to which the electrode is inserted.

4 $$R=C\rho {\text{ln}}{K}_{{\text{H}}}/2\pi h.$$

In the given context, ${\text{C}}$ represents the coefficient associated with the heat distribution of the charge, ρ denotes the mean resistivity of the charge across layers, ${{\text{K}}}_{{\text{H}}}$ signifies the multiplier of the electrode's central distance, and ${\text{h}}$ corresponds to the depth at which the electrode is inserted into the charge.

The correlation between the power of the ferroalloy submerged arc furnace and variations in the depth of electrode insertion is as follows:

5 $$P\left({h}_{max}-\Delta h\right)=\left(1-3\frac{\Delta h}{{h}_{max}}+3{\left(\frac{\Delta h}{{h}_{max}}\right)}^{2}-{\left(\frac{\Delta h}{{h}_{max}}\right)}^{3}\right)P\left({h}_{max}\right).$$

**Figure 5:**
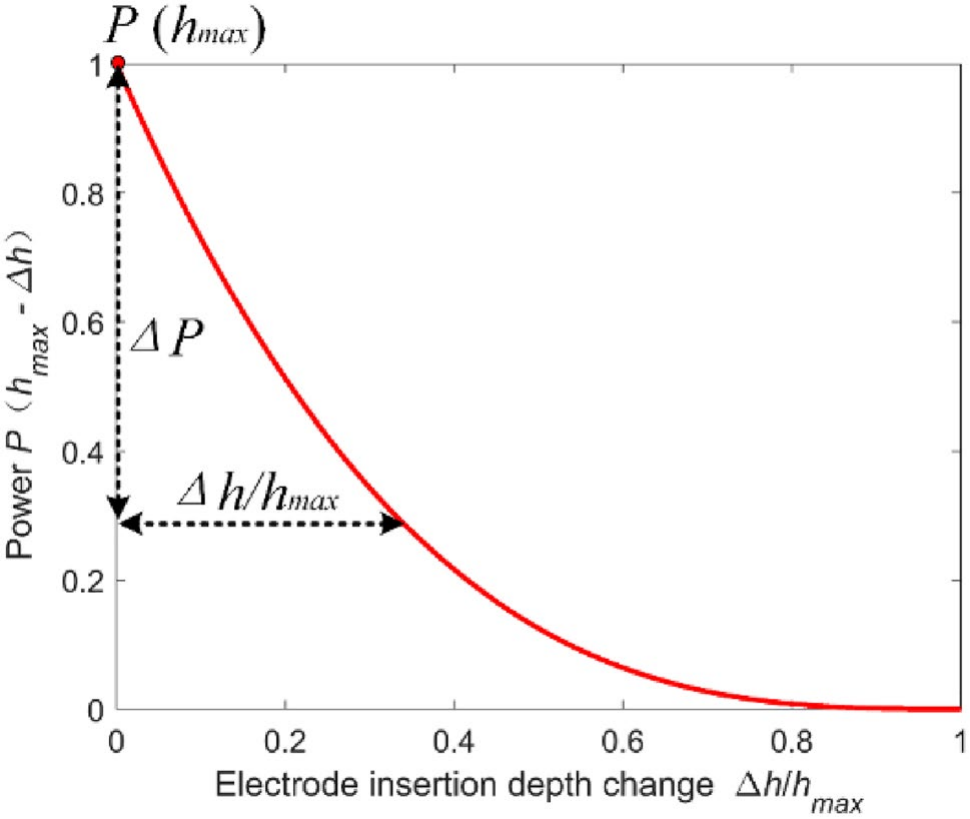
Relation between ferroalloy submerged arc furnace power and electrode insertion depth change.

Consequently, by altering the electrode insertion depth, one can adjust the power of the submerged arc furnace, as depicted in Fig. 5.

### Dynamic prediction model

#### LSTM network for dynamic renewable generation prediction

**Figure 6:**
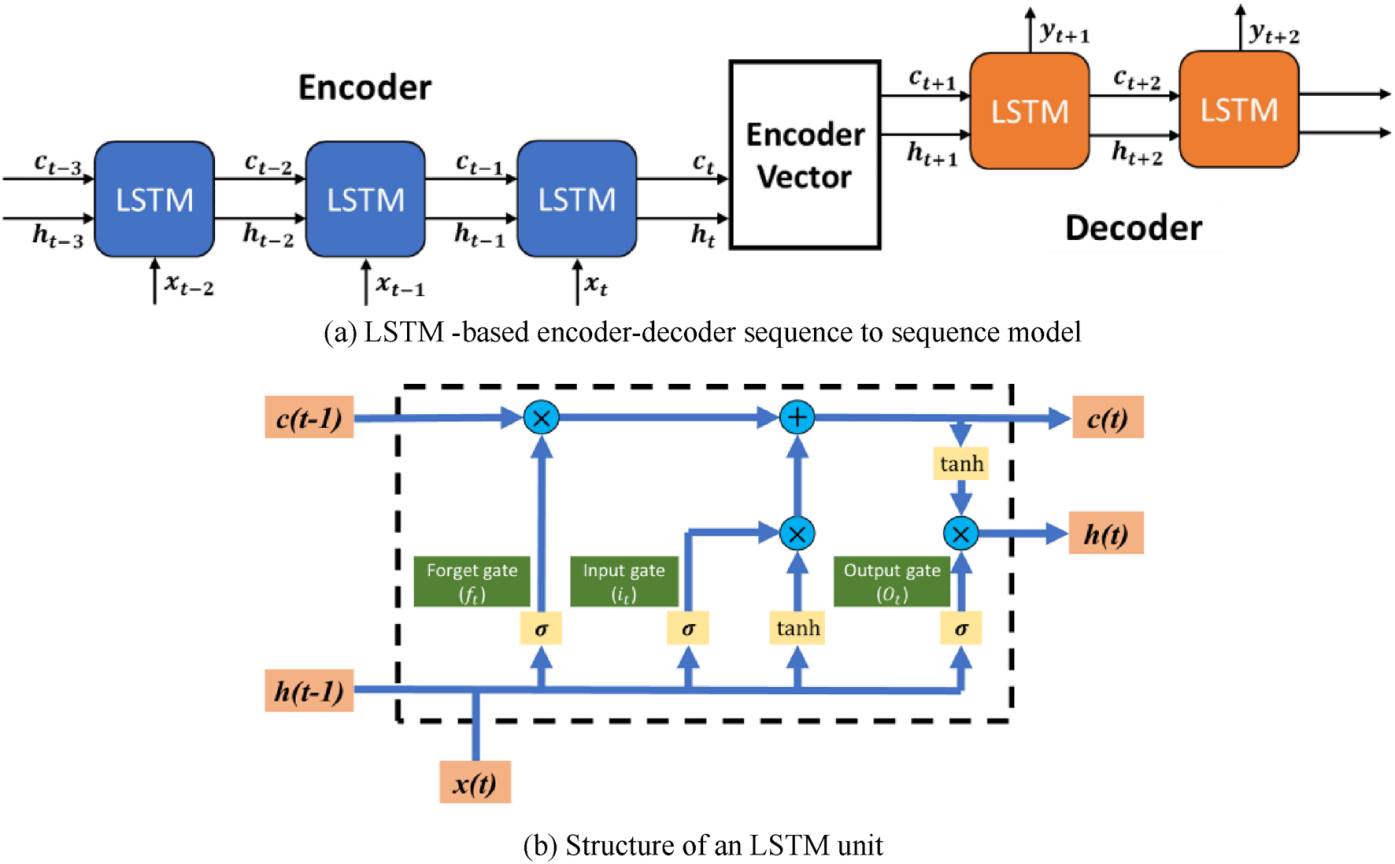
(**a**) LSTM-based encoder-decoder sequence to sequence model and (**b**) Structure of an LSTM unit.

Considering the uncertain renewable generation, a prediction function should be added in the renewable system to facilitate the pricing decision-making process. In this subsection, the LSTM-based sequence-to-sequence model (see Fig. 6a) is formulated to predict future renewable output. This model includes three parts, encoder, encoder vector and decoder, aiming to map a fixed-length input with a fixed-length output where the length of the input and output may differ. The LSTM network is a variant of the standard recurrent neural network (RNN)^19^. By substituting LSTM units (see Fig. 6b) for the basic hidden neurons in RRN, the LSTM network can deal with the issues caused by gradient vanishing and the explosion of long-term dependencies^20^. As shown in Fig. 6, the LSTM unit includes three kinds of gate controllers, i.e., input gate, forget gate, and output gate, which are mainly used to determine what information should be remembered. The following equations can calculate these three gates:

6 $${i}_{t}=\sigma \left({W}_{ix}{x}_{t}+{W}_{ih}{h}_{t-1}+{b}_{i}\right),$$

7 $${f}_{t}=\sigma \left({W}_{fx}{x}_{t}+{W}_{fh}{h}_{t-1}+{b}_{f}\right),$$

8 $${o}_{t}=\sigma \left({W}_{ox}{x}_{t}+{W}_{oh}{h}_{t-1}+{b}_{o}\right),$$

where $\upsigma$ represents the sigmoid function, whose output is in the range of [0,1], describing how much of information should be let through. ${{\text{W}}}_{{\text{ix}}}$, ${{\text{W}}}_{{\text{ih}}}$, ${{\text{W}}}_{{\text{fx}}}$ , ${{\text{W}}}_{{\text{fh}}}$, ${{\text{W}}}_{{\text{fx}}}$ and ${{\text{W}}}_{{\text{fh}}}$ denote matrices of weights for the input gate, forget gate and output gate. ${{\text{b}}}_{{\text{i}}}$, ${{\text{b}}}_{{\text{f}}}$ and ${{\text{b}}}_{{\text{o}}}$ represent the vectors of biases for these gates. It should be noted that temporal memory is implemented in the LSTM network by switching different gates so as to prevent the gradient vanishing. Therefore, the external inputs of the LSTM unit are the previous cell state ${{\text{c}}}_{{\text{t}}-1}$, the previous hidden state ${{\text{h}}}_{{\text{t}}-1}$ and the current input vector ${{\text{x}}}_{{\text{t}}}$.

Then, an intermediate state ${{\text{C}}}_{{\text{t}}}$ is generated, given as,

9 $${C}_{t}=tanh\left({W}_{cx}{x}_{t}+{W}_{ch}{h}_{t-1}+{b}_{c}\right).$$

Accordingly, the memory cell and hidden state of this LSTM are updated as,

10 $${C}_{t}={f}_{t}*{C}_{t}+{i}_{t}*{C}_{t},$$

11 $${h}_{t}={O}_{t}*{\text{tanh}}\left({C}_{t}\right),$$

where tanh is the nonlinear activation function and the operator $*$ denotes the pointwise multiplication operation for two vectors.

In this work, historical data of renewable are collected and put into the proposed encoder-decoder sequence-to-sequence model, where the LSTM network is used as the training algorithm. As the output of this prediction model, ${{\text{y}}}_{{\text{t}}}, {{\text{y}}}_{{\text{t}}+1}, \dots , {{\text{y}}}_{{\text{t}}+12}$ denotes the predicted future 12-h renewable generation. This predicted information will be fed into the Q-learning process to make a pricing strategy in a rolling-window manner.

### ELM algorithm for electricity price prediction

As a well-studied training algorithm, the extreme learning machine (ELM) algorithm has become a popular topic in the fields of load forecasting^21^, electricity price forecasting^22^, and renewable generation forecasting^23^. Since the input weights and biases of the hidden layer are randomly assigned and free to be tuned further when using the ELM algorithm, some exceptional features can be obtained, e.g., fast learning speed and good generation. To deal with the uncertainties of electricity prices and solar generations, we propose an ELM-based feedforward NN to extract features of these two uncertainties dynamically. Specifically, at each hour, the inputs of the trained feedforward NN are past 24-h electricity price data and solar generation data, and its outputs are the forecasted future 24-h trends of electricity prices and solar generations.

**Algorithm 1:**
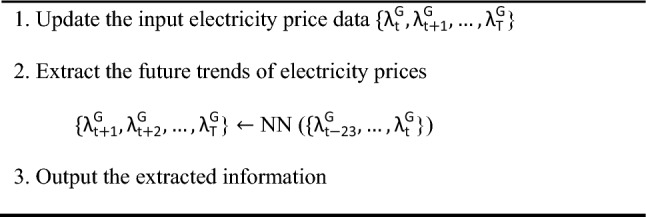
Feedforward NN (Features Extraction).

The trained DFM forecasts future 24-h electricity prices in each time slot, as shown in Algorithm 1, where ${\uplambda }_{{\text{t}}}^{{\text{G}}}$ denotes electricity price in time slot ${\text{t}}$.

**Figure 7:**
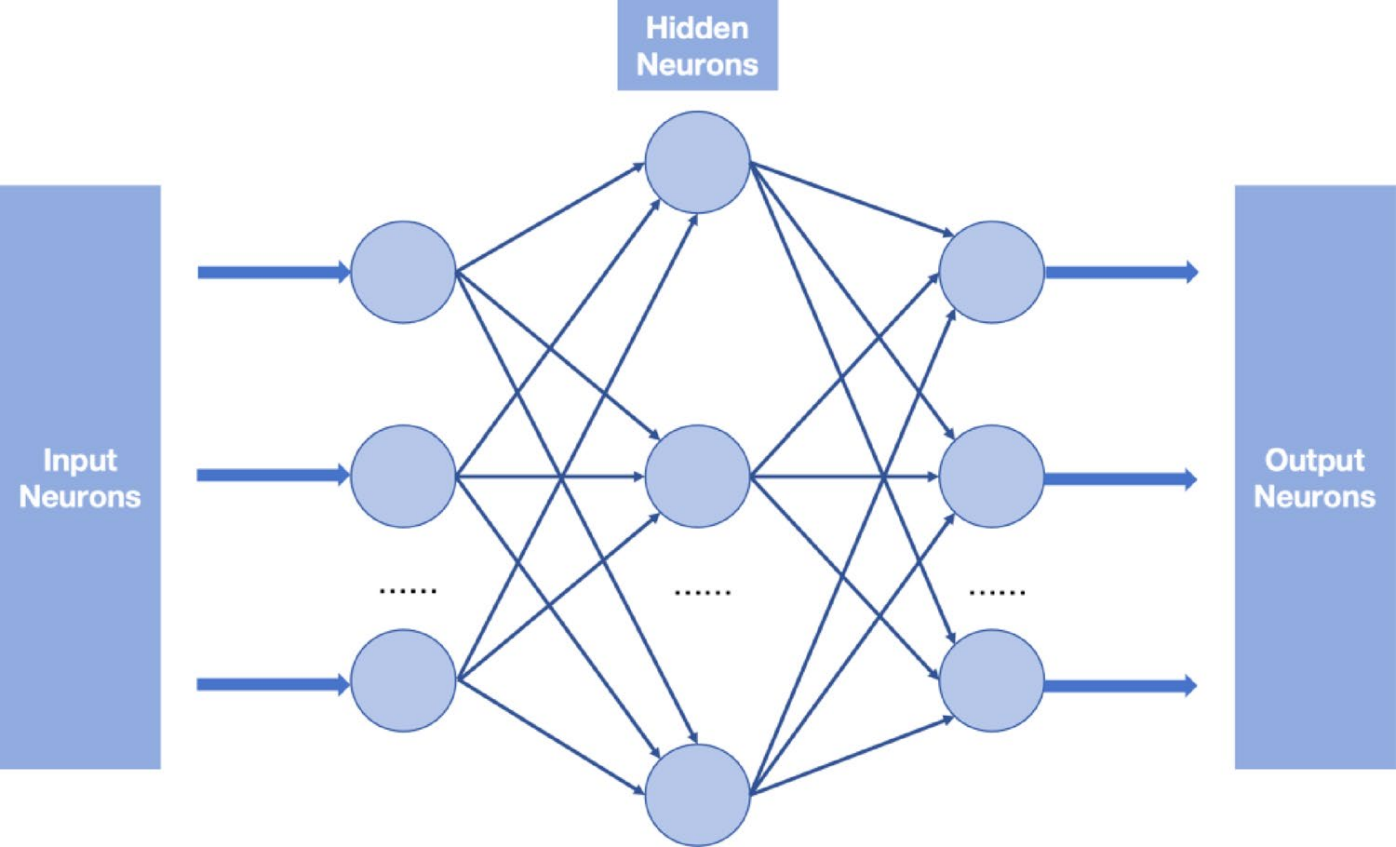
Flowchart of ELM.

The initial training data ${\uplambda }_{{\text{t}}}^{{\text{G}}}$, ${\uplambda }_{{\text{t}}+1}^{{\text{G}}}$ and ${\uplambda }_{{\text{T}}}^{{\text{G}}}$ of this paper are imported, then the hidden layers in the feed-forward neural network are substituted into the neural network nodes, and finally the prediction results of the posterior moments are output as shown in the following Fig. 7.

### Optimization model

In the optimization model proposed in this paper, the objective function comprises multiple sub-functions. This model aims to achieve the optimal power allocation of the surplus output from wind and solar generation by considering various expenditure aspects and satisfying various constraints. The specific mathematical formula is as follows:

12 $$\left\{\begin{array}{l}{{\text{G}}}_{1}=({\uplambda }^{{\text{Sell}}}{{\text{p}}}^{{\text{Sell}}-{\text{Grid}}}-{\uplambda }^{{\text{Buy}}}{{\text{p}}}^{{\text{Buy}}})\\ {{\text{G}}}_{2}=\alpha \cdot {({{\text{P}}}_{{\text{max}}}-{{\text{P}}}_{{\text{SAF}}})}^{2}\\ {{\text{G}}}_{3}=\sum_{{\text{t}}=1}^{{{\text{N}}}_{{\text{T}}}}({{\text{p}}}_{{\text{spot}}}\left({\text{t}}\right)-{{\text{p}}}_{{\text{IBDR}}}({\text{t}}))({{\text{P}}}_{{\text{Kn}},{\text{real}}}\left({\text{t}}\right)-{{\text{P}}}_{{\text{Kn}},{\text{base}}}({\text{t}}))\mathrm{\Delta T}\\ {{\text{G}}}_{4}=\sum_{{\text{t}}=1}^{{{\text{N}}}_{{\text{T}}}}{{\text{p}}}_{{\text{IBDR}}}({\text{t}})({{\text{P}}}_{{\text{Kn}},{\text{clearing}}}\left({\text{t}}\right)-{{\text{P}}}_{{\text{Kn}},{\text{real}}}({\text{t}}))\mathrm{\Delta T}\end{array}\right.$$

13 $$max{F}_{1}=\left({G}_{1}-{G}_{2}+{G}_{3}-{G}_{4}\right),$$

where ${{\text{G}}}_{1}$ represents the gains and losses arising from the process of buying and selling electricity from the grid; ${{\text{G}}}_{2}$ introduces a dissatisfaction factor $\mathrm{\alpha }$ to describe the dissatisfaction cost if the SAF is not at fully rated power, note that $\mathrm{\alpha }$ can be defined by users case by case; ${{\text{G}}}_{3}$ signifies the incentive compensation accrued from the ancillary service market; and ${{\text{G}}}_{4}$ constitutes the penalty incurred due to the discrepancy between the actual power of the ferroalloy submerged arc furnace and the clearing power in the ancillary service market. Furthermore, ${\uplambda }^{{\text{Sell}}}$ is defined as the electricity price for grid sales, subject to temporal fluctuations, altering every fifteen minutes; ${{\text{p}}}^{{\text{Sell}}-{\text{Grid}}}$ is quantified as the electricity quantity sold to the grid from the optimized segment; ${\uplambda }^{{\text{Buy}}}$ is stipulated as the fixed electricity price from the grid; ${{\text{p}}}^{{\text{Buy}}}$ is the volume of electricity procured from the grid; ${{\text{p}}}_{{\text{spot}}}\left({\text{t}}\right)$ is articulated as the electricity price within the spot market; ${{\text{p}}}_{{\text{IBDR}}}({\text{t}})$ is the incentive price for ancillary services; ${{\text{P}}}_{{\text{Kn}},{\text{base}}}({\text{t}})$ is the baseline load of the ferroalloy submerged arc furnace; ${{\text{P}}}_{{\text{Kn}},{\text{real}}}({\text{t}})$ represents the actual power of the ferroalloy submerged arc furnace; and ${{\text{P}}}_{{\text{Kn}},{\text{clearing}}}\left({\text{t}}\right)$ is the clearing power within the ancillary service market; and (13) is the combined objective function.

Subject to:

14 $$\left\{\begin{array}{l}{{\text{P}}}_{{\text{optimal}}}={{\text{P}}}_{{\text{PV}}-{\text{Wind}}}\left({\text{t}}\right)-{{\text{P}}}_{{\text{min}}}\\ {{\text{P}}}_{{\text{optimal}}}={{\text{P}}}_{{\text{optimal}}-{\text{SAF}}}+{{\text{P}}}_{{\text{Sell}}-{\text{Grid}}}\\ {{\text{P}}}_{{\text{Sell}}-{\text{SAF}}}={{\text{P}}}_{{\text{min}}}+{{\text{P}}}_{{\text{optimal}}-{\text{SAF}}}\\ {{\text{P}}}_{{\text{Kn}},{\text{base}}}(t)\le {{\text{P}}}_{{\text{SAF}}}\le {{\text{P}}}_{{\text{max}}}\\ {{\text{P}}}_{{\text{SAF}}}={{\text{P}}}_{{\text{Sell}}-{\text{SAF}}}\\ {{\text{P}}}_{{\text{PV}}-{\text{Wind}}}\left({\text{t}}\right)={{\text{P}}}_{{\text{Sell}}-{\text{Grid}}}+{{\text{P}}}_{{\text{Sell}}-{\text{SAF}}}\end{array}\right.,$$

wherein ${{\text{P}}}_{{\text{optimal}}}$ denotes the excess power available after fulfilling the baseline power demands of the SAFs, ${{\text{P}}}_{{\text{PV}}-{\text{Wind}}}\left({\text{t}}\right)$ encapsulates the aggregated electricity output from both photovoltaic power stations and wind farms at a given temporal node, ${{\text{P}}}_{{\text{min}}}$ is the stipulated minimum power threshold for the SAF, precisely 6.33 MW, ${{\text{P}}}_{{\text{max}}}$ signifies the peak power capacity of the SAF, capped at 10.17 MW, ${{\text{P}}}_{{\text{optimal}}-{\text{SAF}}}$ represents the quantum of the optimize ${{\text{P}}}_{{\text{optimal}}}$ apportioned to the SAF, whereas ${{\text{P}}}_{{\text{Sell}}-{\text{Grid}}}$ corresponds to the fraction of the optimized ${{\text{P}}}_{{\text{optimal}}}$ designated for grid sales, ${{\text{P}}}_{{\text{Sell}}-{\text{SAF}}}$ designates the cumulative power allocated to the SAF, ${{\text{P}}}_{{\text{SAF}}}$ is indicative of the SAF's current operational power, and ${{\text{P}}}_{{\text{Sell}}-{\text{Grid}}}$ stands for the aggregate power dispatched to the grid.

## Case study

### Prediction of renewable energy

The renewable energy dataset for network training is collected from the the Global Energy Forecasting Competition 2014^24^, which can be publicly accessed online. The dataset covers 12 numerical weather prediction (NWP) variables and the hourly renewable power output measured from 1st Apr 2012 to 1st Jul 2014 at three neighboring renewable plants in Australia. In this case, we only use the renewable power output observed in site 1 for model construction, integration of NWP information and the neighbored measurements is beyond the scope of this work, since historic samples are enough to establish the forecasting model on a rolling basis. Before learning, the measurements at night (7:00 pm–7:00 am) are removed, the data from 1st Apr 2012 to 1st Apr 2014 is used for model training, and the rest is for prediction. The settings of the adopted encoder-decoder LSTM network are listed in Table 2.

**Table 2:** Summary of training settings of LSTM network for renewable generation prediction.

Network	Hyperparameter	Value/Function
Encoder	Encoder length	36
	Layers	1
	Hidden states	200
	Kernel_regularizer	0.001
	Activation function	Relu^25^
Decoder	Decoder length	12
	Layers	1
	Hidden states	200
	Activation function	Relu
	Kernel_regularizer	0.001
	MLP layers	1
	MLP activation function	Tanh^26^
Others	Epochs	100
	Batch size	64
	Loss function	Mean squared error^27^
	Optimizer	Nadam^28^

**Figure 8:**
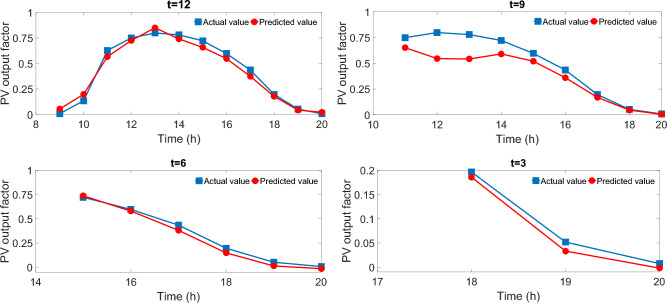
Prediction performance with different time steps.

The predictive skill of the well-trained LSTM network concerning different look-ahead horizons is shown in Fig. 8. Based on this predicted information, a sequence of optimal actions can be selected by the Q-learning agent with the consideration of the trade-off between the current reward and future reward by setting the discount factor. However, only the action for a current hour is executed. Therefore, a relatively large perdition error will have a minor effect on our result. It should be noted that taking into account the NWP information and making a dynamic intraday adjustment on the day-ahead forecast can further improve the forecasting accuracy, which would benefit the reinforcement learning-based decision-making process on finding the near-optimal solutions, this will be investigated in our future work.

## Prediction of price

This study uses real-world data to train our proposed feedforward NN. The hourly data on electricity prices from January 1, 2017, to December 31, 2018 lasting 730 days are collected from PJM^29^. After a number of accuracy tests, the trained feedforward NN for electricity price data consists of three layers, i.e., one input layer with 24 neurons, one hidden layer with 40 neurons and one output layer with one neuron. The number of training episode is 50,000.

**Figure 9:**
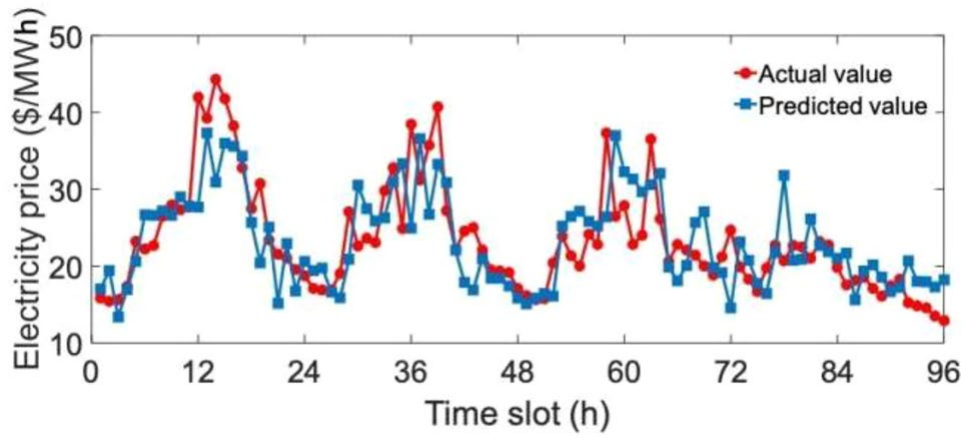
Comparison of the actual and predicted electricity prices on January 1–4, 2019.

Figure 9 shows the performance of the proposed feedforward NN for extracting features of electricity prices. The blue line represents the extracted future values and the red line indicates the actual values. It can be observed that extracted trends of electricity prices are generally similar to actual ones, though small errors can be observed from some time slots. Therefore, the proposed feedforward NN can generate accurate and reasonable forecasting values, which can benefit the following decision-making process for energy consumption scheduling.

### Optimal parameters

**Figure 10:**
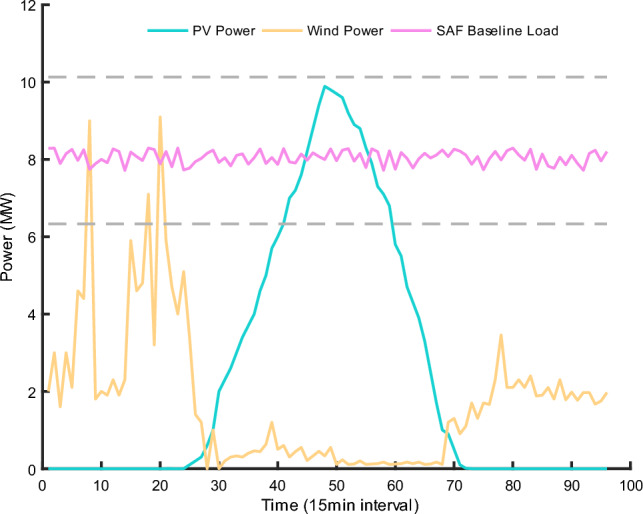
Distributed PV system output curve, wind power absorption curve, and furnace baseline load curve.

Recently developed SAFs are predominantly semi-enclosed or entirely enclosed, featuring low-profile hoods. In regions marked by ethnic diversity, frontier landscapes, and socio-economic challenges, the capacity of these SAFs typically exceeds 12.5 MVA. Consequently, an SAF with a 12.5 MVA capacity has been chosen for analysis, with its associated parameters presented in Table 3. The electrical characteristics of such a furnace can be deduced as follows: The secondary voltage of the transformer varies between 126 and 162 V, with the SAF's designated voltage being 152 V. The standard current for this furnace is 50.30 kA, with a maximum surge up to 62.75 kA. Power metrics for this SAF are delineated as 7.65 MW (nominal), 6.33 MW (minimum), and 10.17 MW (peak). At the same time, Fig. 10 shows the output curve of the distributed PV system, the wind energy absorption curve and the base load curve of the furnace.

**Table 3:** SAF’s parameters.

SAF capacity	12.5 MVA
Transformer secondary voltage range	126–162 V
SAF designated voltage	152 V
Standard current	50.30 kA
Maximum current	62.75 kA
Power (Nominal)	7.65 MW
Power (Minimum)	6.33 MW
Power (Peak)	10.17 MW

Considering the risk associated with uncertain parameters, this article contains a significant number of such uncertainties. Moreover, when these parameters vary, the optimized insertion depth values will be influenced to a certain extent. With regard to corresponding safety concerns, it is generally believed that the electrode is in the most ideal and secure position when the insertion depth is within the range of (0.60 ~ 0.70) times the electrode diameter D. Assuming that the maximum power of the submerged arc furnace is 10.17 MW when the electrode insertion depth is 0.72 m (= 0.7D), based on the relationship between electrode insertion depth and power, the minimum electrode insertion depth is determined to be 0.61 m ($=({{{\varvec{h}}}_{{\varvec{m}}{\varvec{a}}{\varvec{x}}}\cdot (\frac{{{\varvec{P}}}_{{\varvec{m}}{\varvec{i}}{\varvec{n}}}}{{{\varvec{P}}}_{{\varvec{m}}{\varvec{a}}{\varvec{x}}}})}^\frac{1}{3}$). This risk is explicitly incorporated into the optimization code as a constraint to prevent safety risks associated with uncertain parameters.

**Figure 11:**
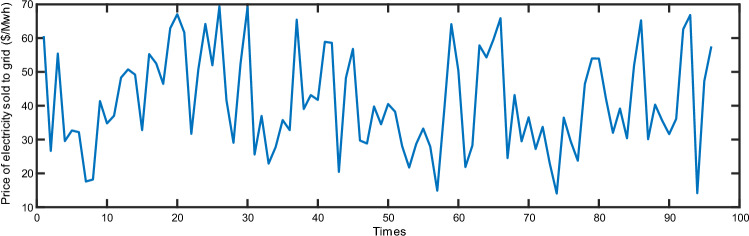
Tariffs for sale.

**Figure 12:**
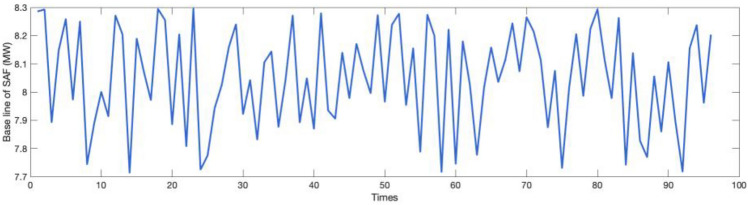
Power Baseline for SAF.

The simulated selling price of electricity in this study varies over time, as illustrated in Fig. 11, fluctuating between 13.8 $/MWh and 69.4 $/MWh. The power baseline used for optimization by SAF is depicted in Fig. 12, hovering around 8 MW. Concurrently, the fluctuation range for the electricity spot market price is 6.3 $/MWh to 83 $/MWh. Within this discussion, the transmission and distribution fee and the government fund are pivotal components. In this paper, both the transmission and distribution fee and the government fund are set at a fixed rate of 14.8 &/MWh; the market rewards price for auxiliary services is set at 27.7 $/MWh. The distributed PV system on-grid pricing is set at 42.75 $/MWh, and the grid electricity selling price is provisionally set at 122.2 $/MWh. Detailed pricing can be found in Table 4.

**Table 4:** Case study specifications for the electricity market.

Electricity market price	Value ($/MWh)
Tariffs for sale	13.8 ~ 69.4
spot prices	6.3 ~ 83
transmission and distribution fee and government fund	14.82
The market rewards price for auxiliary services	27.78
distributed PV system on-grid pricing	42.75

## Results of the proposed planning model

**Figure 13:**
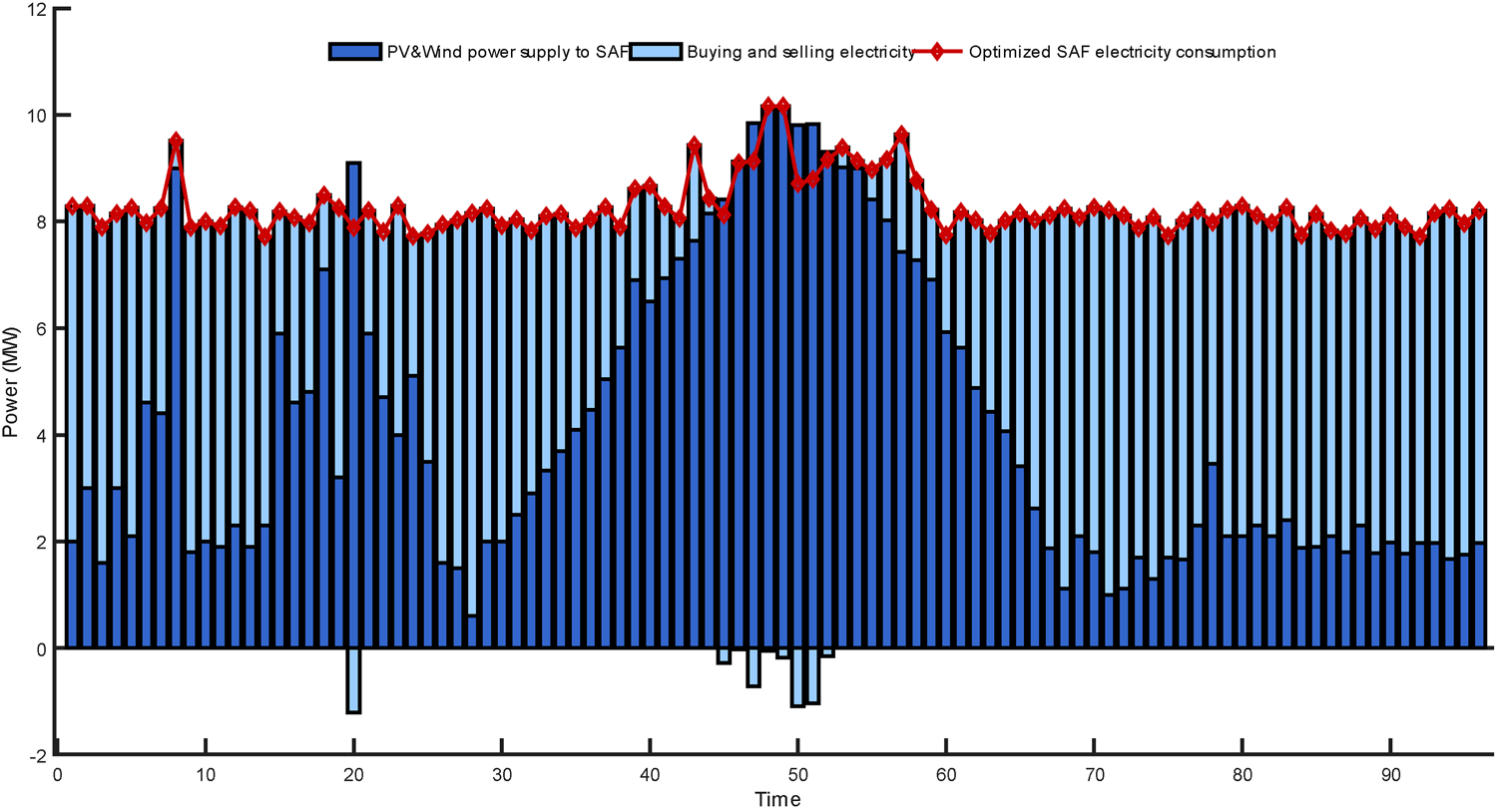
Composition of Submerged Arc Furnace energy consumption in each period (α = 250).

Figure 13 provides a detailed presentation of the energy consumption of the optimized SAF, the allocation of wind and solar power generation, and the electricity transactions with the grid. As illustrated in Fig. 13, within 24 h, the output from wind and solar energy largely satisfies the power demands of SAF. While there are moments when electricity needs to be purchased from the grid, SAF can still effectively absorb a substantial portion of the electricity produced by distributed energy sources. This further proves that, in an electrical network integrated with intelligent energy terminals, utilizing appropriate optimization strategies can significantly enhance the absorption rate of renewable energy sources and reduce energy wastage.

**Figure 14:**
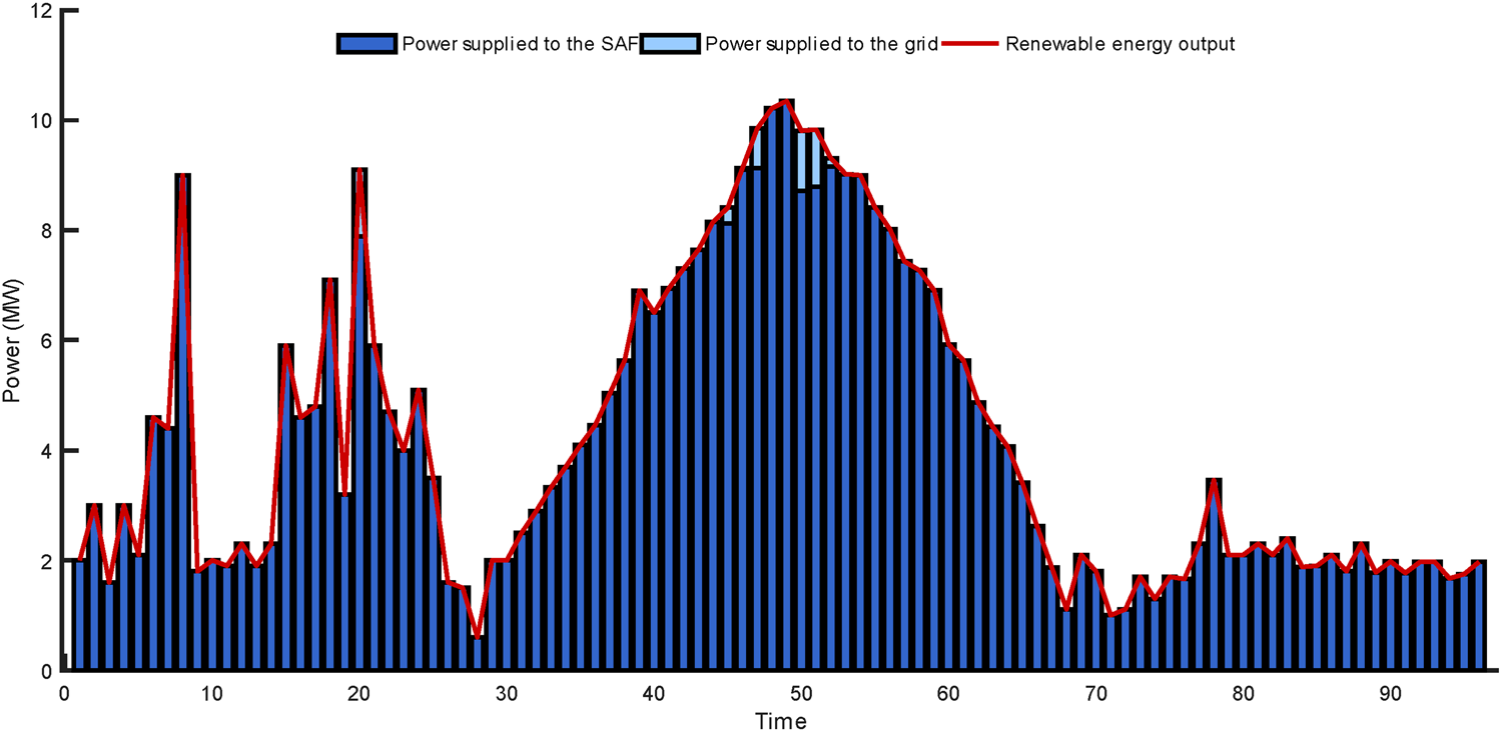
Renewable Allocation (α = 250).

The allocation of renewable energy sources and the generation of electricity from renewable energy sources are given in Fig. 14. As shown in Fig. 14, the renewable energy farm designed here tends to supply the electricity it produces to the SAF system rather than to the grid. Therefore, the SAF's consumption of sustainable energy can be facilitated by integrating the Intelligent energy terminal control system and the prediction model proposed in this paper.

It is widely known that wind and solar power generation are constrained by meteorological conditions, with their output fluctuating by changes in wind speed and solar intensity. Such fluctuations could potentially result in instability in the grid's voltage and frequency, leading to a series of issues, including: (1) deterioration in power quality; (2) increased complexity in grid operational dispatch; (3) electricity price volatility; (4) heightened demand for energy storage. However, by employing the energy-sharing model proposed in this study, SAF's effective absorption of wind and solar energy can significantly mitigate the challenges posed by the intermittency of these sources, thereby enhancing the electrical grid's stability.

The cost data of the optimized time node is shown in Table 5. It can be seen that in the case of multi-constraint optimization, sometimes there is a certain income in the power grid electricity transaction, and sometimes a certain amount of power needs to be spent to purchase electricity to meet the power demand of SAF. At the same time, due to the absorption of distributed energy generation, the market will have certain rewards and fines for such auxiliary services. As indicated in the Table 5 below, after the adaptation of the optimization model proposed in this paper, a total loss recovery of 1906.84 USD per day can be achieved during the optimized time period.

**Table 5:** Optimised values for each objective function (α = 250).

Time node	8	18	20	39	40	41	42	43	44	45	46
G1 ($)	− 7.70	− 251.16	166.52	− 281.87	− 349.29	− 272.23	− 187.07	− 196.12	− 63.72	7.21	42.73
G2 ($)	39.14	74.96	108.26	70.14	68.15	94.33	93.83	41.58	77.57	90.92	52.35
G3 ($)	262.39	223.90	195.90	228.44	230.36	207.00	207.41	259.32	221.50	209.84	246.70
G4 ($)	77.18	65.85	57.62	67.19	67.75	60.88	61.00	76.27	65.15	61.72	72.56
F1 ($)	− 130.88	186.95	161.72	− 214.61	− 277.56	− 234.72	− 169.14	− 63.35	− 10.77	31.81	150.34
Time node	47	50	51	52	53	54	55	56	57	58	59
G1 ($)	148.88	188.34	182.23	64.87	4.07	20.01	− 58.28	− 129.28	− 230.74	− 227.48	− 271.93
G2 ($)	51.28	66.45	63.33	50.35	43.01	51.08	56.78	50.17	35.71	64.28	103.16
G3 ($)	247.89	232.01	235.11	248.94	257.56	248.11	241.89	249.14	266.88	234.17	199.88
G4 ($)	72.91	68.24	69.15	73.22	75.75	72.97	71.14	73.28	78.50	68.87	58.79
F1 ($)	258.94	263.87	264.78	177.10	133.46	130.53	39.15	− 16.63	− 83.93	− 147.06	− 248.39

### Sensitivity analysis of the dissatisfaction factor

**Figure 15:**
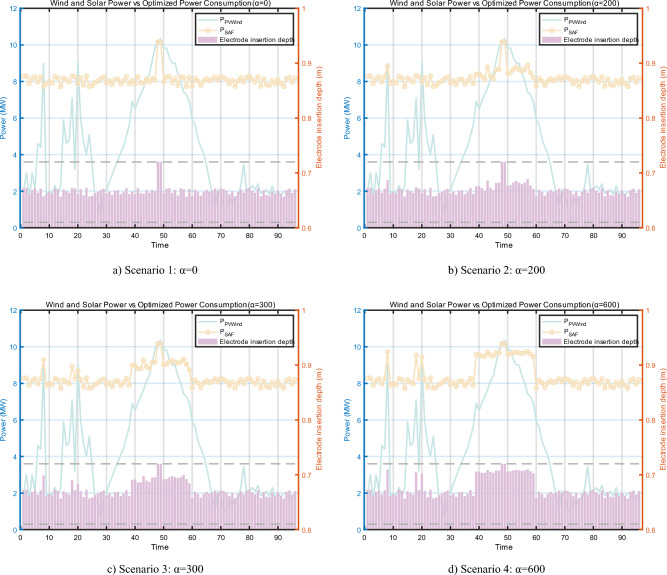
Optimisation results for scenarios with different values of α.

Figure 15 illustrates the optimization outcomes juxtaposed with the electrode insertion depths across four distinct scenarios, characterized by varying α values: Scenario 1 (α = 0), Scenario 2 (α = 200), Scenario 3 (α = 300), and Scenario 4 (α = 600). An inspection of Fig. 15 reveals that the electrode insertion depths consistently lie between 0.61 m and 10.17 m, suggesting that these depths are within a reasonable range.

The light intensity is nearly negligible during the early morning interval, specifically from time node 0 to time node 30. However, the prominence of wind power during nocturnal hours facilitates the harnessing of more wind energy. This results in the emergence of optimized time nodes at 8, 18, and 20. Conversely, during daylight hours, from time node 30 to time node 70, the SAF power optimization closely mirrors the oscillations in renewable energy. Certain time nodes, such as 48 and 49, exhibit power values that approach the peak permissible for SAF, compelling the SAF to operate at its maximum power level.

As the value of α escalates, the power metric of SAF starts to deviate from the SAF baseline. This observation indicates that for higher operational power requirements in the SAF system, it is beneficial to modulate the value of α to exert finer control over power outcomes.

**Figure 16:**
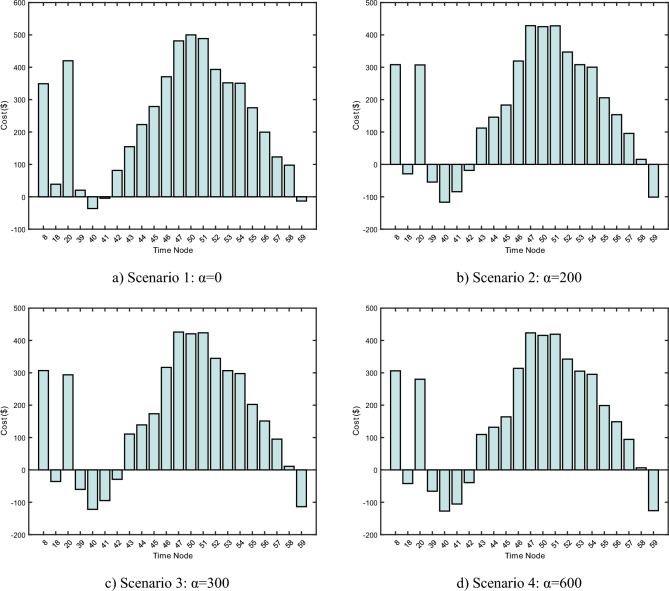
Cost with different values of α.

In Fig. 16, the nodal expenditures are depicted across different optimization scenarios which correlate with varied α values, as per the model proposed in this study. Analogous to Fig. 15, four distinct scenarios are considered with α values set at 0, 200, 300, and 600. Significant optimization outcomes are observed during daylight hours, largely attributed to the escalation in PV power. Specifically, time nodes from 43 to 47 and 50 to 58 yield positive financial outcomes, attributed to the incentives procured from participation in the ancillary market. Conversely, nighttime periods, as seen in time nodes like 8, 18, 20, 39 to 43, are marked by an absence of PV power. The pronounced wind variability during these periods results in fluctuating wind power outputs. Such fluctuations occasionally lead to an inability to secure profits from the ancillary market incentives and notable losses. A comparative analysis of the four scenarios reveals a trend: as the value of α increases, the daytime profitability diminishes, and the nighttime losses intensify.

Consequently, while elevating the value of α can yield a higher SAF power, especially under conditions of elevated operating power requirements in the SAF system, prudent calibration of α is imperative. An excessively high α might lead to nighttime losses surpassing daytime profits, whereas an unduly low α might produce optimization results that fall short of the anticipated SAF power benchmarks. Therefore, determining the α value necessitates judicious adjustments tailored to specific operational conditions to ensure desirable outcomes.

### Compared with existing research

**Figure 17:**
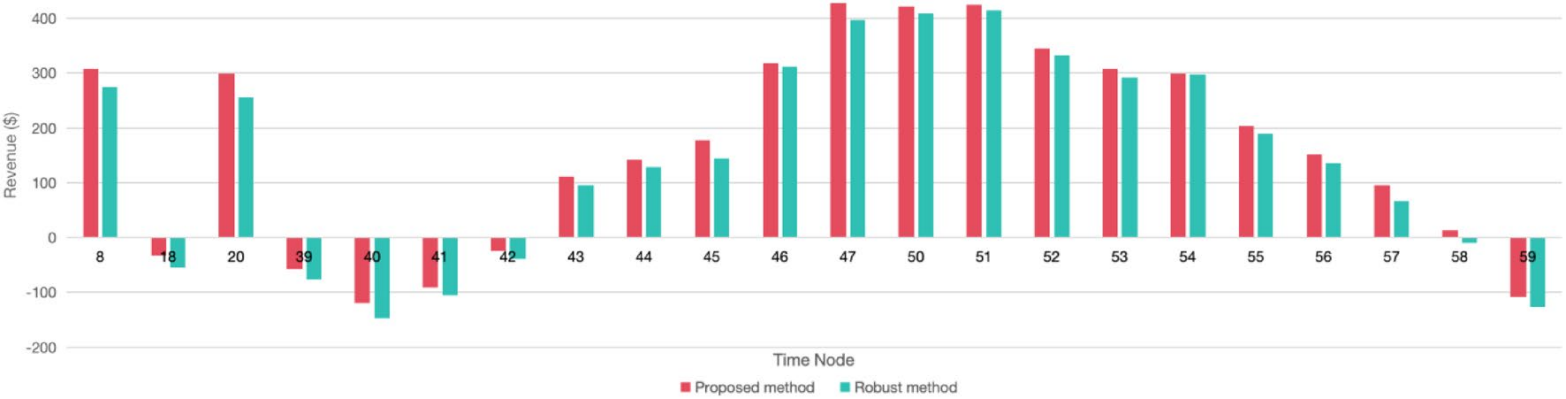
The revenue of different optimization methods (α = 250).

Robust optimization is a widely adopted optimization technique, predicated on the principle of formulating models based on the least favorable conditions, reflecting a conservative approach while emphasizing safety. In this subsection, we have employed robust optimization to enhance our existing model and have illustrated the outcomes in Fig. 17. This figure presents a comparative analysis of the costs associated with our optimization method versus those obtained via robust optimization, all predicated on a uniform dissatisfaction coefficient. It is discernible from Fig. 17 that the profits yielded by our optimization strategy generally surpass those of the robust optimization approach; correspondingly, the losses incurred by our method are also consistently lower than those resulting from robust optimization. Notably, at the 58th time node, robust optimization resulted in a deficit, whereas our approach, harnessing LSTM and ELM for the prediction of baseline data, achieved profitability. Table 6 lists the total revenue of the SAF system under the two optimisation methods, the proposed method and the robust method, and it is clear from the comparison that the total daily revenue of the system for the proposed method is higher than that of the robust method by $426.24. These empirical findings substantiate the superiority of our optimization algorithm over extant research methodologies, striking a balance between ensuring safety and economic viability.

**Table 6:** Total revenue of the two methods.

	Total revenue
Proposed method ($)	3610.34
Robust method ($)	3184.10

## Conclusion

This paper presents an innovative method for harmonizing Submerged Arc Furnace (SAF) systems with renewable energy sources within modern intelligent energy terminals. Employing a dual-phase approach, it first utilizes data-driven predictions to navigate the complexities of renewable energy fluctuations and variable electricity market rates. Specifically, an LSTM network is implemented for the predictive analysis of renewable energy output, showcasing its proficiency in addressing the intricacies of fluctuating energy sources. Concurrently, the ELM model is harnessed for forecasting electricity prices, providing valuable insights into the dynamic market scenario. Following the predictive phase, an optimization model is applied for pinpoint accuracy in SAF dispatching. Local renewable energy consumption is maximized through strategic liaisons between intelligent energy terminals, demand-side mechanisms, and load management infrastructure. Our case study, anchored on a 12.5 MVA SAF system interfaced with 12 MW renewable generators in a fluctuating electricity market, underscores the model's capability to slash operational costs and amplify renewable energy use. By comparing the results with the robust optimisation, the optimisation in this paper outperforms the robust optimisation and is more economical with safety. Such strides forward promise enhanced grid resilience and decreased renewable energy wastage (Supplementary Information).

### Supplementary Information

Supplementary Information.

## Data Availability

All data is available upon request. Please contact Dr Xu Xu (xu.xu02@xjtlu.edu.cn) if someone wants to request the data from this study.
